# Preoperative Embolization of Glomus Tumors: Role, Effectiveness, and Complications

**DOI:** 10.3390/jcm13195905

**Published:** 2024-10-03

**Authors:** Rana Garayzade, Jakob Leicht, Niklas Eckardt, Sven Koscielny, Thomas E. Mayer

**Affiliations:** 1 Department of Neuroradiology, Institute of Diagnostic and Interventional Radiology, University Hospital Jena, 07747 Jena, Germany; jakob.leicht@med.uni-jena.de (J.L.); niklas.eckardt@med.uni-jena.de (N.E.); t.e.mayer@med.uni-jena.de (T.E.M.); 2 Department of Otorhinolaryngology, University Hospital Jena, 07747 Jena, Germany; sven.koscielny@med.uni-jena.de

**Keywords:** glomus tumor, embolization, bleeding

## Abstract

**Purpose:** Paragangliomas represent a surgical challenge due to their hypervascularization. The preoperative selective embolization of these tumors significantly decreases intraoperative blood loss. However, the literature on preoperative embolization in glomus tumors is limited. The aim of this study is to contribute additional evidence regarding the role of preoperative embolization, as well as to evaluate risks and complications in the treatment of glomus tumors. **Methods:** A retrospective evaluation of all the embolizations of glomus tumors from 2009 to 2023 was conducted. The primary outcome parameter was the rate of devascularization after embolization and the occurrence of significant perioperative hemorrhages. The secondary outcome was embolization-related complications. **Results:** Twenty-one embolizations in 20 patients were investigated in the study. In 43% of the cases more than 90% devascularization was achieved by embolization, while in the remaining cases, 80 to 90% devascularization was reached. In one case (5%), significant perioperative bleeding after embolization occurred. In one case (5%), a symptomatic complication occurred periinterventionally due to the brief dislocation of the coaxial and microcatheter into the internal carotid artery (ICA), which led to fresh punctate DWI lesions on the subsequent MRI. No patients developed nerve palsy following embolization. **Conclusions:** The preoperative embolization of glomus tumors can lead to significant tumor devascularization and a reduction in perioperative bleeding, with a low complication rate.

## 1. Introduction

Glomus tumors, or paragangliomas of the head and neck, are rare tumors that arise in adulthood from paraganglia or glomus cells within the carotid glomus, vagus nerve, middle ear, or jugular foramen [1]. They are highly vascular and complete surgical resection is usually curative, although it can be complicated by dramatic blood loss requiring transfusions [2]. The use of preoperative embolization in treatment has proven promising in preparing patients for surgery by reducing complication rates and intraoperative bleeding [3,4,5]. The benefits for the surgeon also include improved visualization and facilitated dissection, allowing for complete tumor excision [6].

The literature on preoperative embolization in glomus tumors is limited, and many studies have gaps regarding embolization techniques, materials used, intervention outcomes, and complications [7,8]. Embolization should be performed exclusively by neuroradiology specialists who possess knowledge of all anatomical vascular variants and anastomoses. Understanding the arterial supply of cranial nerves can help in selecting embolic agents that allow for maximum occlusion while minimizing the risk of complications. Risks associated with embolization include the potential migration of embolic material into the cerebral circulation, leading to stroke [8]. A major complication of any embolic material is migration into adjacent non-tumor vessels, which can lead to neurological deficits [9,10].

In summary, there is still a lack of comprehensive, evidence-based studies on this topic. This retrospective study may contribute additional evidence regarding the role of preoperative embolization, as well as the risks and complications, in the treatment of glomus tumors. This, in turn, can help optimize neuroradiological embolization in this group of rare tumors.

## 2. Methods

### 2.1. Data Collection

This retrospective study was approved by the local ethics committee. All the patients who underwent embolization of glomus tumors in our center between 2009 and 2023 were included.

### 2.2. Interdisciplinary Approach

Tumor embolization was almost always a preoperative measure and was conducted as an interdisciplinary decision in collaboration with colleagues from otorhinolaryngology (head and neck surgeons, ENT). The patients were counseled and informed by neuroradiology. The presence of an MRI, or in exceptional cases, a CT with a contrast agent, was required. All the embolizations were conducted under general anesthesia. A total of 3 senior physicians specializing in interventional neuroradiology were involved in the procedure. Additionally, 3 senior ENT surgeons, highly specialized in ENT surgery were involved in treating this type of tumor. In one session, directly prior to embolization, diagnostic angiography was performed with the selective visualization of the internal and external carotid arteries to depict possible anastomoses. One multimorbid patient in our cohort decided against surgery and underwent only embolization. All the other patients underwent surgical tumor resection within six days after embolization. All the patients had regular follow-up assessments with clinical and ultrasound examinations.

### 2.3. Embolization Procedure

All the syringes and other materials that came into contact with the embolization mixture remained on a separate table. Initially, a diagnostic angiography of the tumor feeders via the external carotid artery was performed. An 80-cm 6 French femoral sheath (Cook) was used to gain access to the femoral artery via a standard Seldinger technique. We generally administered 3000 units of heparin following femoral access in all the patients. For embolization, an Envoy 5F coaxial catheter (with Terumo-35) was recommended, as it passed through the external branches very well. The embolization was done using a 0.010 inner lumen microcatheter. This was guided to the tumor with a 0.014-inch microguidewire (e.g., Traxess-14), aiming for a selective yet comprehensive position that did not extend deep into the tumor distally, ensuring blood flow towards the tumor. Embolization particles were mixed with contrast agent in a 1:1 to 1:5 concentration. For embolization, the valve with the flush on the microcatheter was removed. A 1 mL syringe loaded with the embolization mixture mixed with contrast agent was attached and slowly injected under fluoroscopic guidance. Using contrast agent further helped in recognizing the flow direction and avoiding reflux into parenchymal vessels. Special attention was paid to the collateral vessels from external arteries to the internal carotid or vertebral arteries (e.g., maxillary artery via the Vidian artery to the internal carotid artery in the carotid canal area, via the inferolateral trunk to the cavernous segment of the internal carotid artery, from the occipital artery to the C1 branch of the vertebral artery). Under fluoroscopic control, the mixture of contrast material and microspheres was slowly injected until stagnation of the contrast agent in the feeding artery was accomplished. After the cessation of tumor inflow and the confirmation of reflux, the microcatheter was flushed with saline or contrast agent to clear embolic material from the microcatheter. The flush of the coaxial catheter was stopped, and the microcatheter was withdrawn, allowing the coaxial catheter to bleed slightly to prevent particle displacement. Then, the flush of the coaxial catheter was restarted, and a control series was performed. If there were additional tumor feeders, the procedure was repeated accordingly. Finally, a control angiography was performed for an overview. A balloon blockade of the ICA was not used due to the risks of clamping, thrombosis, thromboembolism, or dissection.

### 2.4. Angiographic Evaluation

DSA images were reviewed after the completion of embolization, and the percentage of reduction was quantified according to the remaining tumor blush before and after the intervention.

### 2.5. Outcomes

The primary outcome parameter was the rate of devascularization after embolization and the occurrence of a significant perioperative hemorrhage. A significant hemorrhage was defined as any bleeding necessitating blood transfusion, the significant impairment of tumor visualization perioperatively, or surgical revision.

The secondary outcome was complications during the intervention, which included nerve paralysis, stroke, vascular injuries, and bleeding.

### 2.6. Statistics

Descriptive statistics were used to summarize patient characteristics and outcomes. Categorical variables were expressed as numbers and percentages.

## 3. Results

### 3.1. Patient Population

Twenty-one embolizations of 20 patients were investigated in the study.

Baseline presentation included sex, age, tumor location and side, presenting symptoms, and tumor size. The median age was 57 years, with an interquartile range (IQR) of 17 years. The female-to-male ratio was 1.9:1. Nine patients had glomus jugulare tumors, three had glomus tympanicum tumors, fourhad glomus vagale tumors, and fourhad glomus caroticumtumors (as shown in Table 1). The mean tumor size, using the greatest linear diameter on preoperative MRI imaging, was 36 mm. The most common initial presenting symptom was hearing loss in 12 cases (57%).

### 3.2. Post-Treatment

The patient was visited by the interventionalist after embolization and before surgery. Further postoperative controls were the responsibility of the ENT specialist.

### 3.3. Embolization Outcomes

All the patients (excluding one patient who did not undergo surgery after embolization) underwent preoperative tumor embolization a median of 1 day (IQR 1) before surgery, with a mean of 2 days before surgery. The ascending pharyngeal artery was the most common feeder in the 19 embolizations (90%). Other feeders included the occipital artery (in 10 cases), posterior auricular artery (in six cases), and the maxillary, superficial temporal, and meningeal arteries (each in one case).

Eleven embolizations were performed with particles only, six with particles plus coils, and two with particles plus Glubran (Histoacryl). One embolization was performed with Glubran (Histoacryl) only, and one with Onyx 18 only (as shown in Table 2).

### 3.4. Primary Outcome: Effectiveness of Embolization

**Figure 1:**
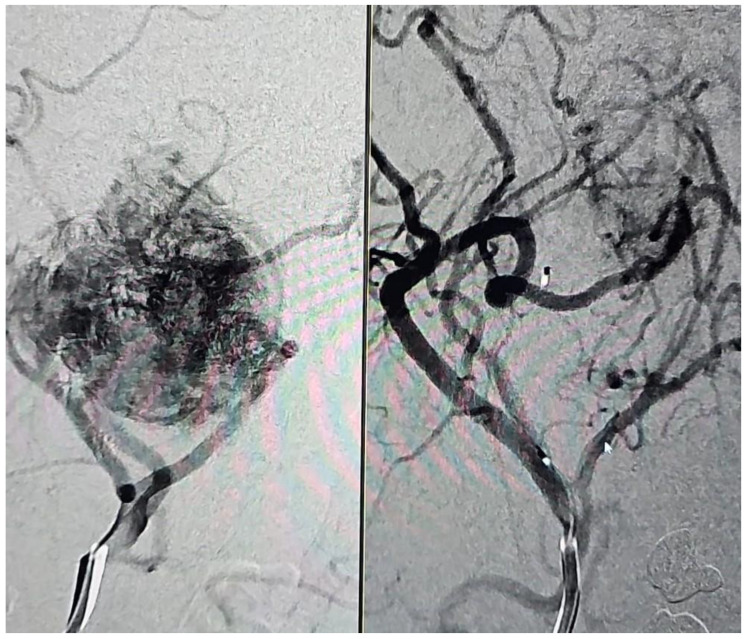
Patient with glomus vagale tumor, pre- and post-embolization (Number 10).

**Figure 2:**
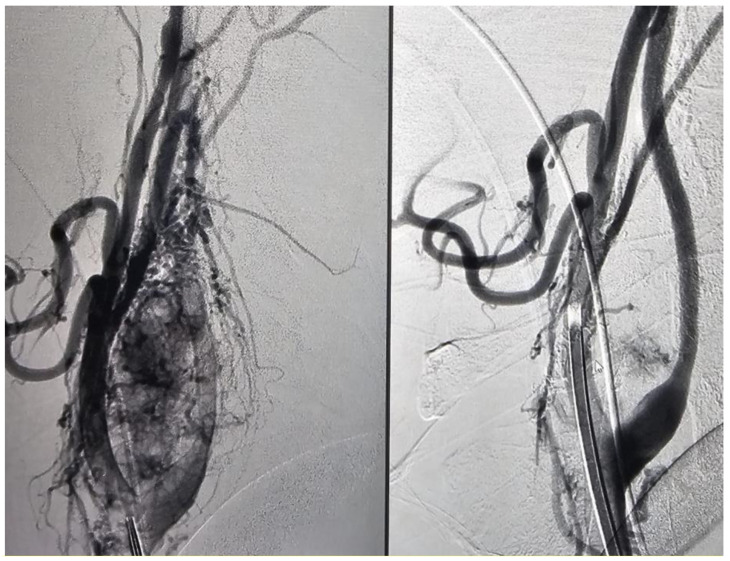
Patient with glomus carotid tumor, pre- and post-embolization (Number 2).

In 43% (9/21) of the cases, 90 to 100% devascularization was achieved by embolization (Figure 1), while in the remaining cases, 80 to 90% devascularization was reached (Figure 2). In one case (1/21, 5%), significant perioperative bleeding occurred, necessitating a blood transfusion and surgical revision.

### 3.5. Peri-Interventional Complications

In one case (1/21, 5%), the patient experienced a symptomatic complication during the intervention, involving the dislocation of the microcatheter into the internal carotid artery (ICA). In this case, three branches of the external carotid artery (ECA) with an elongated vascular course were embolized. Due to the ECA elongation, the microcatheter snapped into the ICA, leading to the transient washout of particles. The dislocation was detected immediately, and embolization was stopped. However, the patient suffered from hemiparesis, and fresh punctate DWI lesions were observed on subsequent MRI. The symptoms fully regressed by day six, followed by tumor resection surgery on the same day.

### 3.6. Recurrence

Two patients in our cohort experienced recurrences. One patient did not undergo preoperative embolization due to an AV shunt demonstrated in the diagnostic angiography, which made safe embolization impossible without the risk of embolization material displacing into the lungs. Due to significant perioperative bleeding, the tumor could not be optimally removed. Subsequently, it progressed rapidly and aggressively, leading to significant tumor growth within one year after surgery. Diagnostic angiography showed significantly altered vascular anatomy after the first operation, with strong feeders from the maxillary artery, which were subsequently embolized, and the tumor was completely surgically removed. No recurrence was observed in the following six-year follow-up.

Another patient experienced a recurrence four years after embolization and subsequent surgery. This patient was then re-embolized and re-operated. Interestingly, the feeder vessel originated from a vessel that did not show tumor blush during the first embolization and was not embolized then.

### 3.7. Embolization without Operation

One multimorbid patient in our cohort decided against surgery and underwent only embolization with annual ultrasound follow-ups by ENT specialists, showing no size progression and remaining asymptomatic for 9 years.

## 4. Discussion

Superselective embolization of glomus tumors performed by an experienced neuroradiologist can produce effective and safe tumor devascularization, providing the surgeon with significant advantages such as decreased blood loss, less need for a blood transfusion, improved visualization and ease of dissection, and increased confidence in achieving complete tumor excision [5,6]. In our cohort, we had one patient who could not be embolized, and due to significant perioperative bleeding, an optimal resection was not possible.

However, it remains unclear which embolization material is most effective for glomus tumors while minimizing embolization-related complications. Embolization carries the risk of the potential migration of embolization material into the cerebral circulation, leading to stroke, especially in the presence of anastomoses between the internal and external carotid systems or flow reversal from the tumor’s arterial blood supply [11].

Understanding arterial anatomy reveals important clinical implications for the embolization of glomus tympanicum and jugulare tumors. The petrosal (arising from the middle meningeal artery) and stylomastoid arteries (arising from the occipital or posterior auricular artery) supply the facial nerve, while the inferior tympanic artery (a branch of the ascending pharyngeal artery) supplies Jacobson’s nerve (a branch of the glossopharyngeal nerve) and Arnold’s nerve (a branch of the vagus nerve). Therefore, there is a higher risk of nerve paralysis when embolizing these arteries, particularly when using liquid embolic agents [12].

If an occipito–vertebral anastomosis exists, temporary occlusion proximal to the anastomosis is imperative for safe embolization [12]. We used coils for permanent occlusion proximal to the anastomosis between the occipital artery and the vertebral artery in 6 patients from our cohort.

### 4.1. Embolization with Onyx

The advantages provided by Onyx in terms of the penetration of intratumoral vessels must be weighed against the risk of cranial neuropathy [13]. Embolization with Onyx can result in cranial nerve palsy post-embolization in up to 28% of cases, by devascularizing at least 60% of the initial tumor blush [10]. Although good results have been reported with percutaneous embolization of carotid paragangliomas using Onyx [14,15], paragangliomas located near the skull base may not be suitable for this treatment due to the difficulty of access. Transarterial embolization with Onyx for carotid paragangliomas is not recommended in one study [10].

We used liquid embolic agents in three patients—once with Onyx and twice with Histoacryl (Glubran)—without complications and with good results. However, compared to particle embolization, the overall course was somewhat more complicated according to studies. Therefore, we now refrain from using liquid embolic agents for the intra-arterial embolization of glomus tumors.

### 4.2. Particle Embolization

Particle embolization allows for more controlled embolization by utilizing varying particle sizes to limit penetration into anastomotic branches that serve the vasa vasorum of nerves [16]. In one study of 29 patients, embolization with polyvinyl alcohol (PVA) particles (75% of cases using a particle size of 250–350 microns) resulted in a greater than 50% reduction in tumor blush in 86.2% of cases, with no new or worsening cranial neuropathy, although 31% required intraoperative blood transfusions [16].

In another study, 38 patients underwent selective arterial embolization of their paragangliomas using PVA particles ranging in size from 100 to 1000 microns. Post-embolization angiography revealed an average decrease in the blood flow to the tumor of 75% [17].

The major disadvantage of PVA particles is their tendency to aggregate, causing unexpected proximal vessel occlusion and potential catheter blockage. For this reason, small particle sizes should be used, although they are presumably less steerable. Through distal penetration, this can also lead to the occlusion of vasa nervorum and deficits in cranial nerves. We used microspheres that, even at a size of 250 to 500 microns, have good distal penetration but do not lead to the occlusion of vasa nervorum. PVA particles may also accumulate in the catheter hub, risking nontarget embolization when flushed [18].

Due to the irregular shape of PVA particles and the associated major issues of catheter occlusion and proximal vessel occlusion without good vessel penetration, spherical and calibrated PVA particles, such as Bead Block, were developed. These problems are, however, significantly reduced with microspheres like Embospheres and the more precisely calibrated Embozenes [19].

Embozene Microspheres are available in prefilled 20 mL or 2 mL syringes, suspended in transport solution or contrast medium, in a range of sizes suitable for embolic therapy. These microspheres, suspended in contrast material, are spherical, smooth, flexible, easily compressible, and precisely calibrated; these physical properties enhance the ease of use without the occurrence of catheter blockage [20]. Embozene has a unique polyphosphazene coating (commercial name: Polyzene-F^®^, Alta Biomed, 6070 Corte Del Cedro, Unit A, Carlsbad, CA 92011) that acts as an antithrombogenic and anti-inflammatory material [19,21].

Compared to Embospheres, Embozene offers precisely calibrated particles by size, which is evidently important to avoid complications. In a study involving the embolization of meningiomas using Embospheres (100–300 and 40–120 μm), 6.5% neurological complications and 3.2% hemorrhagic complications were reported [22]. In another study using Embozene 400, there were no neurological or hemorrhagic complications, compared to the 8.3% overall complication rate using small PVA particles (45–150 μm) in the historical cohort [20].

In recent years, through our in-house experience with the use of particles in neuroradiological interventions, we have concluded that we have had better experiences with Embozene particles in sizes 250 and 400, without complications directly related to the embolization material.

A study aimed at determining the histopathologic changes occurring in glomus tumors after embolization with PVA particles showed that the embolization particles undergo fragmentation and 30% of embolized vessels undergo partial revascularization at 9 to 16 days of post-embolization [23]. Therefore, it is recommended to perform surgery within the first 8 days. The timing of post-embolization surgery is crucial due to potential inflammatory and ischemic reactions caused by the use of polyvinyl alcohol particles for embolization (150 to 300 μm), which complicate tissue dissection, necessitating close collaboration between the embolizer and the surgeon [5]. Whether this also applies to embolization with Embospheres is not known. Our embolizations were performed within the first 8 days.

In summary, in our experience, Embozene microspheres particles of 250–500 µm provide sufficient distal penetration to achieve good devascularization of the tumor, but without excessive distal penetration, as seen with Onyx or smaller-sized PVA particles, which can lead to the occlusion of the vasa nervorum of the cranial nerves [8]. On the other hand, PVA particles of approximately the same size show noticeably worse penetration, resulting in relevant perioperative bleeding in 31% of cases, compared with only one case (5%) in our study [16].

Important limitations of this study include the retrospective study design and the small patient sample size.

## 5. Conclusions

The preoperative embolization of glomus tumors can lead to significant tumor devascularization and reduction in perioperative bleeding, with a minimal complication rate.

## Figures and Tables

**Table 1:** Baseline characteristics.

Patient Number	Sex	Age at Embolization	Tumor	Side	Symptoms	Tumor Max. Size
1	F	64	G. jugulare	Right	Hearing loss	31 mm
2	M	50	G. caroticum	Left	Hearing loss	56 mm
3 No embolization	F	49	G. jugulare	Left	Hearing loss, tinnitus	31 mm
3 Recurrence	F	50	G. jugulare-Recurrence	Left	Recurrence by follow-up after one year without symptoms	25 mm
4	M	31	G. tympanicum	Left	Hearing loss, tinnitus	43 mm
5	F	72	G. vagale	Left	Palpable/fungating mass, vocal cord paralysis	45 mm
6	M	67	G. jugulare	Right	Hearing loss	52 mm
7	F	43	G. jugulare	Right	Hearing loss, tinnitus	35 mm
8	F	58	G. jugulare	Left	Tinnitus	21 mm
9	M	52	G. jugulare	Left	Hearing loss, tinnitus	31 mm
10	F	50	G. vagale	Right	Palpable/fungating mass	45 mm
11	M	80	G. caroticum	Right	Neck mass, dysphagia, night sweats	22 mm
12	F	65	G. tympanicum	Left	Otalgia, otorrhea, hearing loss	34 mm
13	F	62	G. jugulare	Left	Hearing loss, tinnitus, dizziness	44 mm
14	M	66	G. jugulare	Right	Hearing loss, tinnitus	40 mm
14 Recurrence	M	69	G. jugulare- Recurrence	Right	Recurrence by follow-up without symptoms	20 mm
15	F	31	G. caroticum	Right	night sweats	25 mm
16	F	32	G. tympanicum	Left	Hearing loss, tinnitus, initial suspected cholesteatoma in CT-scan with attempted surgery in peripheral clinic with bleeding	37 mm
17	F	53	G vagale	Right	Dysphagia, palpable mass	45 mm
18	F	74	G. caroticum	Right	Palpable mass	26 mm
19	M	66	G. vagale	left	Dizziness	58 mm
20	F	69	G. jugulare	Left	Hearing loss, tinnitus, initial suspected cholesteatoma in CT-scan with attempted surgery in peripheral clinic with bleeding	20 mm

**Table 2:** Embolization characteristics.

Patient Number	Operation on the Day after Embolization	Arterial Feeders	Embolization Material	Complication of Embolization	Extent of Devascularization (%)	Significant Perioperative Bleeding	Last Control (Years after Embolization)
1	Day 1	Occipital a., ascending pharyngeal a., posterior auricular a.	Particle 500, Coils	No	>90	No	13
2	Day 1	Ascending pharyngeal a., posterior auricular a.	Particle 500, Histoacryl (Glubran)	No	>90	No	10
3	No embolization	Ascending pharyngeal a.	No Embolization	N.A.	N.A.	Yes (significant perioperative bleeding, an optimal tumor resection was not possible)	(1)
3 (Recurrence)	Day 1	Maxillary a.	Histoacryl (Glubran)	No	80	No	6
4	Day 1	Occipital a., ascending pharyngeal a., posterior auricular a.	Particle 250, Histoacryl (Glubran)	No	>90	No	5
5	Day 1	Ascending pharyngeal a.	Particle 500	No	90	No	9
6	No operation	Occipital a., ascending pharyngeal a.	Particle 500, Coils	No	>90	n.a.	9
7	Day 2	Occipital a., ascending pharyngeal a.	Particle 250	No	80	No	7
8	Day 6	Occipital a., ascending pharyngeal a.	Particle 250/500	Yes, dislocation of the microcatheter, displacement of particles into the ICA	>90	No	9
9	Day 2	Ascending pharyngeal a.	Particle 250, Coils	No	>90	No	5
10	Day 2	Occipital a., ascending pharyngeal a., superficial temporal a.	Particle 500	No	85	No	5
11	Day 2	Ascending pharyngeal a.	Onyx 18	No	90	No	5
12	Day 5	Occipital a., ascending pharyngeal a.	Particle 500, Coils	No	90	No	5
13	Day 5	Occipital a., ascending pharyngeal a.	Particle 250, Coils	No	>90	No	5
14	Day 4	Ascending pharyngeal a., posterior auricular a.	Particle 250	No	80	No	Recurrence after 4 years
14 (Recurrence)	Day 1	Occipital a.	Particle 250, Coils	No	85	No	1
15	Day 2	Ascending pharyngeal a.	Particle 400	No	>90	No	2
16	Day 1	Medial meningeal a.	Particle 400	No	>90	No	2
17	Day 1	Occipital a., ascending pharyngeal a.	Particle 400	No	90	No	2
18	Day 1	Ascending pharyngeal a., posterior auricular a.	Particle 400	No	80	No	2
19	Day 1	Ascending pharyngeal a.	Particle 400	No	80	Yes (significant bleeding with operative revision)	2
20	Day 2	Ascending pharyngeal a., posterior auricular a.	Particle 250/400	No	80	No	1

## Data Availability

The raw data supporting the conclusions of this article will be made available by the authors on request.
